# Offset loading in hexagonal bar deadlift: a ‘stealth’ strategy for acutely modulating neuromuscular activation asymmetry and enhancing acute jump performance

**DOI:** 10.3389/fphys.2026.1768828

**Published:** 2026-03-13

**Authors:** Shuwei Chen, Sen Yang, Xiubing Zhang, Jiahui Ye, Shanxin Feng, Wenbai Huang, Weibao Liang

**Affiliations:** 1 Institute of Athletic Training Science, Capital University of Physical Education and Sports, Beijing, China; 2 Institute for Sport Performance and Health Promotion, Capital University of Physical Education and Sports, Beijing, China; 3 Guangdong Provincial Key Laboratory of Speed Capability Research, Su Bingtian Center for Speed Research and Training, School of Physical Education, Jinan University, Guangzhou, China

**Keywords:** asymmetry index, core stability, motor control, post-activation performance enhancement (PAPE), unilateral training

## Abstract

**Objectives:**

Traditional bilateral training often masks functional imbalances, potentially reinforcing dominance in the stronger limb. This study investigated the acute effects of minor offset loading (2.5% and 5% of 1RM) during the hexagonal bar deadlift (HBD) on neuromuscular activation patterns and subsequent power performance, aiming to explore its efficacy in acutely modulating bilateral activation asymmetry.

**Methods:**

Twelve resistance-trained male athletes with right-side dominance participated in a randomized crossover design. Participants performed HBDs under five conditions: Symmetric, and Offset loading (Left/Right) at 2.5% and 5% intensities. Surface electromyography (sEMG) was recorded from the Erector Spinae (ES), External Oblique (EO), Gluteus Maximus (GM), and Rectus Femoris (RF) bilaterally. Vertical jump height (CMJ) and Rating of Perceived Exertion (RPE) were assessed immediately post-exercise.

**Results:**

Baseline data under the Symmetric condition revealed a neuromuscular activation asymmetry, with significantly higher activation in the dominant (Right) RF and ES (p < 0.05). Offset loading elicited a robust, linear dose-response shift in neuromuscular drive (*R* ^2^ > 0.95). Specifically, shifting 5% of the load to the non-dominant (Left) side shifted the Rectus Femoris Asymmetry Index from −3.85% (Right-biased) to +7.41% (Left-biased), with all participants (100%) showing increased agonist recruitment. The External Oblique displayed an inverse activation pattern, confirming a contralateral stabilizing mechanism. Crucially, while RPE increased with offset magnitude (p < 0.001), loading the non-dominant side significantly potentiated CMJ height by 8.2% (p < 0.001), whereas dominant-side loading yielded no significant power gain.

**Conclusion:**

Minor offset loading acts as a “stealth” stimulus that effectively overrides the body’s default recruitment patterns. By acutely increasing neural drive to the weaker limb and engaging contralateral core stabilizers, non-dominant side offset loading not only acutely shifts the activation bias but also unlocks greater post-activation performance enhancement (PAPE) than traditional symmetric loading. These findings provide a quantitative framework for integrating offset HBD into strength and rehabilitation programs to resolve imbalances without inducing excessive fatigue.

## 1 Introduction

Bilateral strength asymmetry—defined as the inability to produce equal force output between the left and right limbs—is a prevalent phenomenon in competitive athletes, often stemming from limb dominance, sport-specific demands, or previous injury (Bishop et al., 2018; Girard, 2025). While a threshold of functional asymmetry is considered normal, excessive inter-limb imbalances (>10–15%) have been associated with reduced movement efficiency, impaired change-of-direction speed, and an elevated risk of non-contact injuries, particularly to the anterior cruciate ligament (ACL) (Myer et al., 2006; Fox et al., 2023). Consequently, identifying and correcting these neuromuscular imbalances is a primary objective in strength and conditioning and rehabilitation settings.

Traditional bilateral resistance training exercises, such as the back squat and conventional deadlift, are fundamental for developing maximal force and power (Nigro and Bartolomei, 2020; N. Munger et al., 2022). However, the bilateral nature of these movements allows for compensatory mechanisms; the dominant limb often assumes a disproportionate share of the mechanical load, potentially masking or even reinforcing existing asymmetries (Kalata et al., 2020). Unilateral-biased exercises (e.g., Bulgarian split squats) preferentially load one limb but still require bilateral support and may constrain absolute loading due to balance demands (McBride et al., 2010; Zhang et al., 2023). Therefore, a “hybrid” strategy that maintains the high-loading capacity of bilateral exercises while enforcing limb-specific recruitment is warranted.

Offset loading (or uneven loading) has emerged as a promising modality to address this limitation (Ostrowski et al., 2017; Lawrence et al., 2021; Sharp et al., 2022). By redistributing the load on a barbell, an external rotational torque is introduced, necessitating increased neuromuscular drive to maintain postural equilibrium (Munoz-Martel et al., 2019; Sharp et al., 2022). Previous research has primarily focused on the upper body, demonstrating that offset bench press significantly increases muscle activation in the primary movers on the loaded side and enhances core stabilizer recruitment (Ostrowski et al., 2017; Lawrence et al., 2021; Sharp et al., 2022). However, the acute neuromuscular responses to offset loading during closed-kinetic-chain lower body exercises remain underinvestigated. While upper-body findings provide a conceptual basis, extending this paradigm to closed-kinetic-chain lower-body exercises like the deadlift is crucial, as they involve substantially greater multi-joint coordination, ground-based postural control, and axial loading demands. Furthermore, the hexagonal bar deadlift (HBD) offers a unique biomechanical profile—characterized by a more upright torso and reduced lumbar shear stress compared to the straight bar deadlift (Swinton et al., 2011; Camara et al., 2016)—making it an ideal candidate for implementing offset loading with reduced injury risk.

Moreover, recent evidence suggests that targeted high-intensity contractions can induce Post-Activation Performance Enhancement (PAPE), acutely improving subsequent explosive performance (Blazevich and Babault, 2019). Yet, it remains unknown whether the unique neuromuscular demand of offset loading—specifically when targeting the weaker limb—might interact with or optimize the global PAPE effect by uniquely stimulating the weaker neural pathway prior to bilateral execution.

Therefore, the purpose of this study was to investigate the acute effects of minor offset loading (2.5% and 5% of 1RM) during the HBD on bilateral neuromuscular activation patterns and subsequent vertical jump performance. We hypothesized that: (1) offset loading would induce a linear, load-dependent shift in agonist muscle activation toward the loaded side; (2) the contralateral core musculature would exhibit increased activation to counteract lateral flexion and axial rotation moments/torques; and (3) loading the non-dominant limb would be expected to acutely redirect functional asymmetry and influence subsequent countermovement jump (CMJ) height.

## 2 Methods

### 2.1 Experimental approach

This study utilized a randomized, counterbalanced crossover design to investigate the acute neuromuscular and kinetic responses to offset loading during the hexagonal bar deadlift (HBD). To isolate the effects of load distribution from load magnitude, the total system mass was maintained constant at 70% of the subject’s one-repetition maximum (1RM) across all conditions. The independent variable was the load distribution configuration, consisting of five levels: Symmetric (control), Left Offset (2.5% and 5% of 1RM shifted to the left), and Right Offset (2.5% and 5% shifted to the right). Dependent variables included surface electromyography (sEMG) amplitude of bilateral trunk and lower limb muscles, vertical jump performance (countermovement jump height, CMJS), and rating of perceived exertion (RPE).

### 2.2 Subjects

Twelve resistance-trained male university athletes (age: 22.2 ± 1.9 years; height: 180.1 ± 5.1 cm; body mass: 73.8 ± 4.5 kg; HBD 1RM: 150.8 ± 15.9 kg; relative strength: 2.04 ± 0.18 kg kg^-1^) volunteered for the study. An *a priori* power analysis (G*Power 3.1; F-test, repeated-measures ANOVA, within factors) was conducted. Assuming a medium effect size (f = 0.30), an alpha level of 0.05, a power of 0.80, five measurements (conditions), and a conservative expected correlation among repeated measures of 0.5, a minimum sample size of 12 was required. Inclusion criteria were: (1) >2 years of consistent resistance training experience; (2) ability to lift ≥1.5 times body mass in the HBD; (3) right-side limb dominance as determined by the Waterloo Footedness Questionnaire; and (4) no history of musculoskeletal injury in the preceding 6 months. All participants provided written informed consent prior to participation. The study protocol was approved by the Ethics Committee of Capital University of Physical Education and Sports (Approval No. 2025A137) and registered with the Chinese Clinical Trial Registry (ChiCTR2500112689). The study was conducted in accordance with the Declaration of Helsinki.

### 2.3 Procedures

#### 2.3.1 Familiarization and baseline testing

Participants visited the laboratory on two occasions separated by at least 72 h. During the first visit, anthropometric data were collected, and limb dominance was determined. Subsequently, participants performed a standardized 1RM HBD testing protocol following National Strength and Conditioning Association (NSCA) guidelines. After determining the 1RM, participants were familiarized with the specific offset loading conditions to minimize learning effects.

#### 2.3.2 Experimental protocol

On the second visit, participants completed a standardized warm-up (10 min cycling at 140 W followed by dynamic stretching). Participants then performed five sets of HBD (one set per condition) in a randomized order. Each set consisted of five repetitions (for 2.5% offset and symmetric conditions) or three repetitions (for 5% offset conditions) to avoid fatigue accumulation, with a constant load of 70% 1RM. A 5-min passive recovery interval was strictly enforced between sets to allow for phosphocreatine resynthesis and to washout acute fatigue. The offset was created by redistributing specific weight plates (equivalent to 2.5% or 5% of the subject’s 1RM) from one side of the hexagonal bar to the other, ensuring the total lifted mass remained unchanged.

#### 2.3.3 Measurements

##### 2.3.3.1 Surface electromyography (sEMG)

Muscle activation was recorded bilaterally from the Erector Spinae (ES; L3 level), External Oblique (EO), Gluteus Maximus (GM), and Rectus Femoris (RF) using a wireless EMG system (Delsys Trigno Avanti, Delsys Inc., United States) at a sampling rate of 2000 Hz. Skin preparation included shaving and cleaning with alcohol to reduce impedance. Sensors were placed parallel to muscle fibers in accordance with SENIAM guidelines. To normalize EMG data, Maximum Voluntary Isometric Contractions (MVIC) were performed for each muscle group post-warm-up. Three 5-s maximal efforts were recorded with 1-min rest intervals. Raw EMG signals were band-pass filtered (10–500 Hz) and full-wave rectified. The root mean square (RMS) was calculated using a 100-ms moving window. The concentric phase was visually identified and time-synchronized using a 2D video camera recording at 60fps/a linear position transducer attached to the barbell. To avoid exaggeration from transient artifacts, the peak RMS was defined robustly as the highest average activation sustained over a continuous 250-ms window within the concentric phase, rather than an instantaneous spike. This robust peak value was then averaged across repetitions for each set, and normalized to the peak MVIC amplitude (%MVIC) (Camara et al., 2016).

##### 2.3.3.2 Assessment of asymmetry

To quantify the magnitude and direction of neuromuscular imbalance, an Asymmetry Index (AI) was calculated for each muscle group using the following formula:

$$AI\left(\%\right)=\frac{\left(EMG_{Left}-EMG_{Right}\right)}{\left(EMG_{Left}+EMG_{Right}\right)}\times 100$$

Positive values indicate left-side dominance, while negative values indicate right-side dominance.

##### 2.3.3.3 Countermovement jump (CMJS) and RPE

Sixty seconds after completing each HBD set, participants performed two maximal countermovement jumps on a force platform (Kistler 9260AA, Switzerland). The highest jump height, calculated *via* flight time, was recorded to assess the acute Post-Activation Performance Enhancement (PAPE) effect. Immediately post-jump, participants rated their perceived exertion using the standard Borg RPE scale (6–20).

### 2.4 Statistical analyses

Data are presented as Mean ± Standard Deviation (SD). Normality was verified using the Shapiro-Wilk test.

For EMG data, a Linear Mixed-Effects Model (LMM) was employed to account for the nested structure of the data and individual variability. The model included Load Condition (5 levels) and Side (Left vs. Right) as fixed effects, with Subject as a random intercept. This approach offers superior robustness for repeated measures data compared to traditional ANOVA.

For CMJS and RPE, a one-way repeated measures ANOVA was conducted to compare differences across the five load conditions. While the LMM was strictly necessary for EMG data to handle the nested nature of bilateral measurements (Left and Right sides within the same subject), CMJS and RPE provided single, systemic output values per condition, making a standard one-way RM-ANOVA appropriate. Bonferroni-corrected *post hoc* tests were used for pairwise comparisons.

Effect sizes were calculated using Cohen’s d (for pairwise comparisons) or partial eta squared (η_p_^2^) where appropriate. Effect size magnitudes for Cohen’s d were classified as small (0.20), medium (0.50), and large (0.80). For partial eta squared (η_p_^2^), thresholds were set at small (0.01), medium (0.06), and large (0.14). Statistical significance was set at p < 0.05. All analyses were performed using R statistical software (Version 4.5.0).

## 3 Results

### 3.1 Participant characteristics

Descriptive characteristics of the participants are presented in Table 1. All subjects identified as right-leg dominant. The cohort demonstrated a high level of relative strength (HBD 1RM: 2.04 ± 0.18 kg kg^-1^), indicating that they were well-trained and capable of tolerating the prescribed loading protocols.

**TABLE 1:** Descriptive characteristics of the participants (N = 12).

Characteristic	Mean ± SD	Range
Age (years)	22.2 ± 1.9	20–25
Height (cm)	180.1 ± 5.1	172–188
Body Mass (kg)	73.8 ± 4.5	68–82
Training experience (years)	3.8 ± 1.2	2–6
1RM HBD (kg)	150.8 ± 15.9	130–180
Relative strength (1RM/BM)	2.04 ± 0.18	1.8–2.4

1RM, One-repetition maximum; HBD, hexagonal bar deadlift; BM, body mass.

### 3.2 Acute effects on power performance and perceived exertion

A significant main effect of load condition was observed for Countermovement Jump height (CMJS) (p < 0.001, η_p_^2^ = 0.723). As detailed in Table 2, offset loading applied to the non-dominant (Left) side elicited a significant potentiation in subsequent jump performance. Specifically, the Left 5% condition resulted in the greatest jump height (51.76 ± 6.47 cm), representing an 8.2% increase relative to the Symmetric baseline (47.83 ± 5.52 cm; p < 0.001). Conversely, loading the dominant (Right) side (2.5% or 5%) did not result in any significant improvement in CMJS compared to the symmetric condition (p > 0.05).

**TABLE 2:** Acute effects of offset loading on countermovement jump height and rating of perceived exertion.

Condition	CMJ height (cm)	% Change vs. Sym	RPE (6–20)
Symmetric	47.83 ± 5.52	-	12.33 ± 1.30
Left 2.5%	50.30 ± 6.35	+5.2%	14.50 ± 1.51
Left 5%	51.76 ± 6.47	+8.2%	15.58 ± 1.16
Right 2.5%	47.26 ± 5.45	−1.20%	13.50 ± 1.38
Right 5%	47.80 ± 5.39	−0.10%	14.50 ± 1.31

Values are presented as Mean ± SD. CMJ, countermovement jump; RPE, Rating of perceived exertion. Significance relative to Symmetric condition: p < 0.05, p < 0.001.

Regarding subjective exertion, RPE scores increased significantly across all offset conditions compared to the symmetric baseline (12.33 ± 1.30; p < 0.001). This increase followed a dose-dependent pattern, with the Left 5% condition eliciting the highest perceived exertion (15.58 ± 1.16).

### 3.3 Neuromuscular activation patterns (EMG)

**FIGURE 1:**
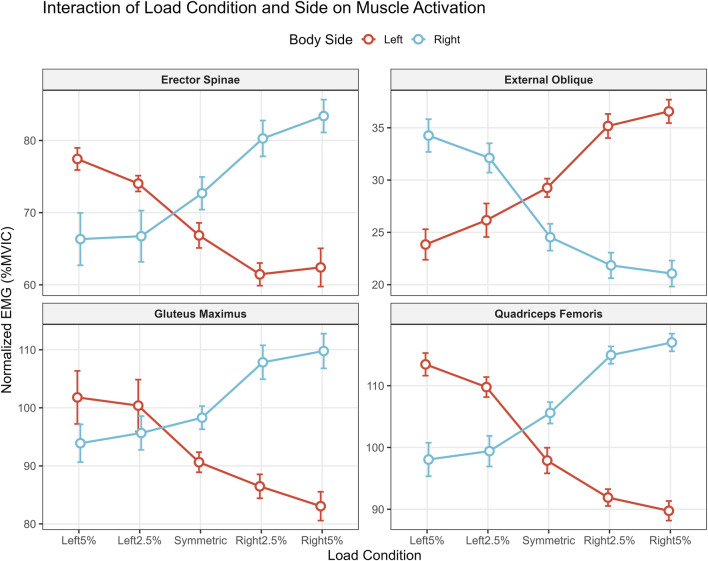
Interaction of load condition and body side on normalized muscle activation. Values are presented as Mean ± Standard Error (SE) to illustrate the dispersion of the sample means. The plots display the significant Condition × Side interaction (p < 0.001) for the Erector Spinae (ES), External Oblique (EO), Gluteus Maximus (GM), and Rectus Femoris (RF). Note the distinct “cross-over” pattern in primary movers (GM, RF), where activation shifts linearly toward the loaded side. In contrast, the External Oblique demonstrates a contralateral activation spike (e.g., right EO increases during left-sided loading), indicating an anti-lateral flexion bracing strategy. Abbreviations: MVIC, Maximum Voluntary Isometric Contraction.

Analysis of raw EMG data (Table 3) revealed a significant Condition × Side interaction (p < 0.001) across all muscle groups (Figure 1).

•Baseline Functional Asymmetry: Under the Symmetric (Control) condition, participants exhibited a natural neuromuscular imbalance. Activation of the Rectus Femoris (RF) and Erector Spinae (ES) was significantly higher on the dominant (Right) side compared to the non-dominant (Left) side (Right RF: 105.6 vs. Left RF: 97.9 %MVIC; p < 0.05).
- Primary Movers (RF & GM): Offset loading induced a rapid shift in neural drive toward the loaded side. In the Left 5% condition, activation of the left RF surged to 113.5 ± 6.4 %MVIC, significantly surpassing both the symmetric baseline and the contralateral (right) side (p < 0.001). A similar pattern was observed in the Gluteus Maximus (GM), where left-side activation increased significantly during left-sided loading.
- Core Stabilizers (EO & ES): The External Oblique (EO) demonstrated a distinct contralateral bracing mechanism. Unlike the primary movers, the EO showed peak activation on the side opposite to the external load (e.g., Right EO activation peaked at 34.3 ± 5.4 %MVIC during the Left 5% condition).

**TABLE 3:** Summary of methodological quality assessment tools and conclusions.

Muscle group	Condition	Left side (Mean ± SD)	Right side (Mean ± SD)	95% CI (left)	95% CI (right)
Erector spinae	Symmetric	66.8 ± 6.0	72.7 ± 7.8^a^	[63.4, 70.3]	[68.3, 77.1]
	Left 5%	77.4 ± 5.3	66.3 ± 12.6	[74.4, 80.5]	[59.2, 73.4]
	Left 2.5%	74.0 ± 3.8	66.7 ± 12.3	[71.9, 76.2]	[59.8, 73.7]
	Right 2.5%	61.5 ± 5.4	80.3 ± 8.6	[58.4, 64.5]	[75.4, 85.1]
	Right 5%	62.4 ± 9.2	83.4 ± 7.9	[57.2, 67.6]	[78.9, 87.8]
External oblique	Symmetric	29.3 ± 3.1	24.5 ± 4.4	[27.5, 31.0]	[22.0, 27.0]
(Core stabilizer)	Left 5%	23.8 ± 5.1	34.3 ± 5.4	[21.0, 26.7]	[31.2, 37.3]
	Right 5%	36.6 ± 3.9	21.1 ± 4.3	[34.4, 38.8]	[18.6, 23.5]
Gluteus maximus	Symmetric	90.6 ± 6.0	98.3 ± 6.9^a^	[87.2, 94.0]	[94.4, 102.2]
	Left 5%	101.8 ± 15.8	93.9 ± 11.2	[92.8, 110.8]	[87.6, 100.3]
	Right 5%	83.1 ± 8.6	109.8 ± 10.4	[78.2, 87.9]	[103.9, 115.7]
Rectus femoris	Symmetric	97.9 ± 7.2	105.6 ± 6.0^a^	[93.8, 102.0]	[102.2, 109.0]
(Primary mover)	Left 5%	113.5 ± 6.4	98.1 ± 9.4	[109.9, 117.1]	[92.7, 103.4]
	Left 2.5%	109.8 ± 5.6	99.4 ± 8.6	[106.6, 113.0]	[94.6, 104.3]
	Right 5%	89.8 ± 5.5	117.0 ± 4.9	[86.6, 92.9]	[114.2, 119.8]

CI, confidence interval; MVIC, Maximum voluntary isometric contraction. Indicates significant difference from Symmetric condition (p < 0.05).

^a^ Indicates significant difference between Left and Right sides at baseline (Symmetric condition), highlighting baseline activation asymmetry.

### 3.4 Quantification of asymmetry correction and individual responses

**FIGURE 2:**
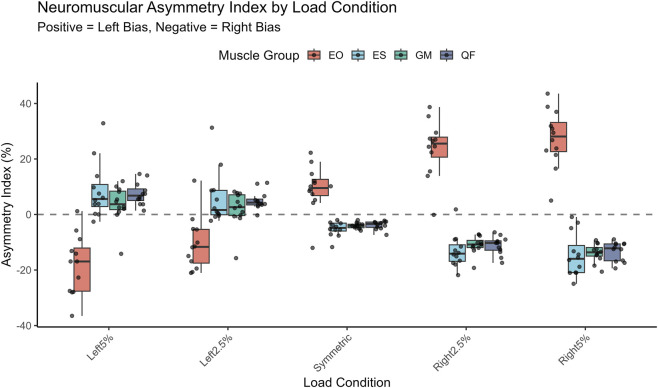
Neuromuscular asymmetry index (AI) across varying load conditions. Box-and-jitter plots represent the distribution of asymmetry for each muscle group. The dashed horizontal line at AI = 0% represents perfect symmetry. Positive values indicate left-side dominance, while negative values indicate right-side dominance. The data reveal a robust linear dose-response relationship (*R* ^2^ > 0.95) for the Erector Spinae, Gluteus Maximus, and Rectus Femoris, where the dominance polarity shifts progressively from right (negative) to left (positive) as the load offset increases. The External Oblique displays an inverse trend consistent with its stabilizing role. The box plots show the median and interquartile range (IQR), with individual data points overlaid to depict biological variability.

The Asymmetry Index (AI) analysis (Table 4) demonstrated a robust, linear dose-response relationship (*R* ^2^ > 0.95 for primary movers) (Figure 2). The introduction of offset loads progressively shifted the dominance polarity. For the Rectus Femoris, the AI shifted from a baseline right-side bias (−3.85%) in the symmetric condition to a significant left-side bias (+7.41%) in the Left 5% condition. This linear correction was also evident in the Erector Spinae and Gluteus Maximus.

**TABLE 4:** Neuromuscular asymmetry index (AI%) across load conditions, illustrating the dose-response shift in dominance.

Muscle group	Right 5%	Right 2.5%	Symmetric	Left 2.5%	Left 5%	Trend
Erector spinae	−14.59 ± 7.81	−13.15 ± 6.02	−4.09 ± 5.18	6.02 ± 9.81	8.53 ± 10.15	Linear shift
External oblique	27.35 ± 10.47	23.75 ± 10.28	9.28 ± 8.55	−10.56 ± 9.56	−18.10 ± 10.85	Inverse linear^a^
Gluteus maximus	−13.86 ± 3.32	−10.93 ± 3.24	−4.04 ± 1.05	1.93 ± 6.55	3.61 ± 6.79	Linear shift
Rectus femoris	−13.21 ± 3.43	−11.16 ± 3.22	−3.85 ± 1.49	5.06 ± 3.32	7.41 ± 4.06	Linear shift

Values are presented as Mean ± SD., The Asymmetry Index (AI) is calculated as (Left - Right)/(Left + Right) × 100. Positive values (+) indicate Left side dominance; Negative values (−) indicate Right side dominance. Indicates significant deviation from the Symmetric baseline (p < 0.05).

^a^ External Oblique displays an inverse trend consistent with contralateral stabilization mechanisms.

**FIGURE 3:**
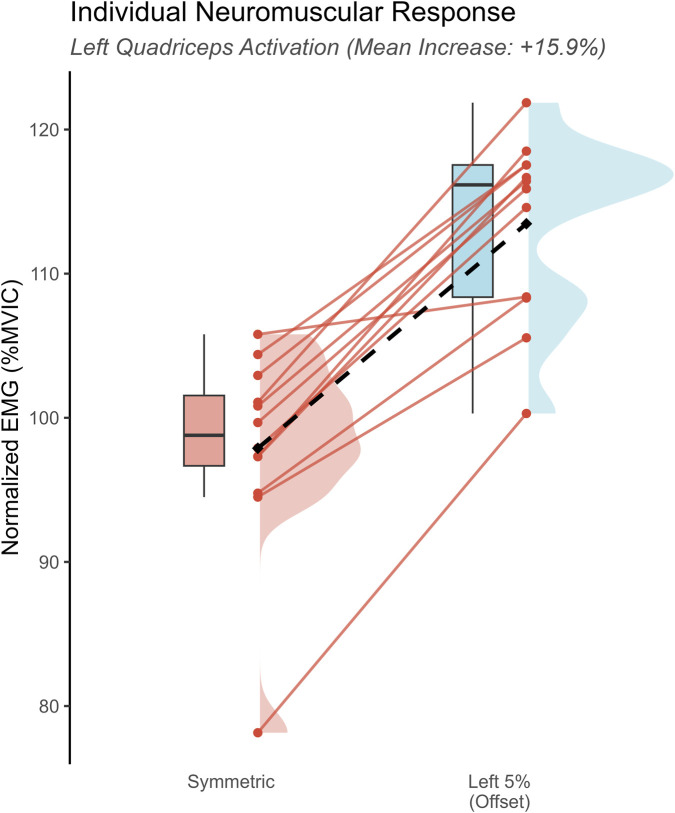
Individual neuromuscular response of the left Rectus Femoris to offset loading. Slopegraph illustrating the acute change in agonist muscle activation for each participant (N = 12) when transitioning from the Symmetric condition to the Left 5% Offset condition. Each line represents a single subject. The background distribution (half-violin and box plot) visualizes the overall population shift. Key Finding: A 100% response rate was observed, with all participants exhibiting increased recruitment of the left Rectus Femoris (mean increase: +15.6 %MVIC). The consistent upward trajectory confirms the robustness of the offset loading stimulus in overriding individual baseline differences.

Individual analysis (Figure 3) revealed that all twelve participants exhibited an acute increase in agonist recruitment (Left RF) when transitioning from Symmetric to Left 5% loading. The magnitude of this neuromuscular upregulation ranged from +2.6% to +22.2% MVIC, indicating a consistent and universal strategy to maintain postural equilibrium regardless of individual baseline strength.

## 4 Discussion

**FIGURE 4:**
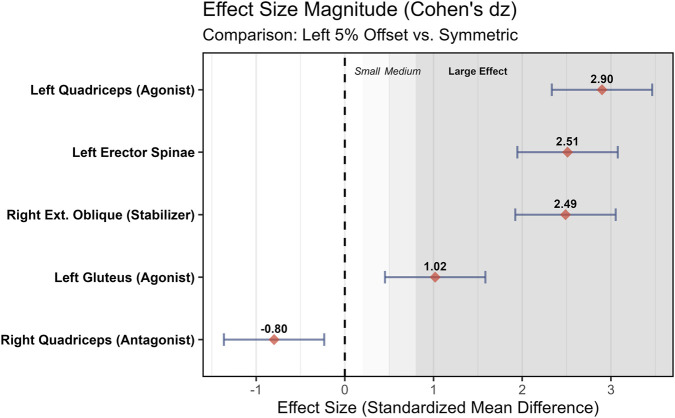
Effect size (Cohen’s dz) of neuromuscular adaptations during Left 5% offset loading compared to Symmetric loading. Forest plot displaying the magnitude of change for key muscle groups. Error bars represent the 95% Confidence Interval (CI). The vertical dashed line at 0 represents no effect. The shaded regions denote effect size thresholds: Small (0.2), Medium (0.5), and Large (0.8). Interpretation: Large effects (dz > 0.8) were observed for the agonist (Left RF, Left GM) and the contralateral stabilizer (Right EO), highlighting the dual mechanism of offset loading: promoting ipsilateral drive while demanding contralateral core rigidity.

**FIGURE 5:**
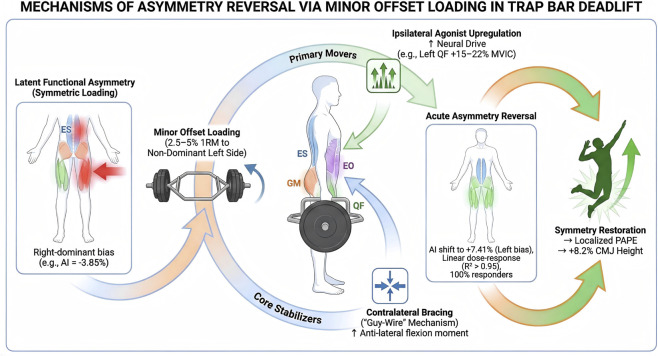
Schematic representation of the “stealth” offset loading strategy: mechanisms of neuromuscular asymmetry correction and acute power potentiation. Baseline Asymmetry: Under symmetric loading, a functional imbalance exists (Right bias), leading to unequal force distribution and potential “force leakage.” The “stealth” stimulus: Introducing a minor (5%) offset to the non-dominant (Left) side creates a rotational torque. This forces the CNS to acutely upregulate neural drive to the ipsilateral agonist (Left RF/GM) while engaging the contralateral core stabilizer (Right EO) via a “guy-wire” mechanism. Outcome: This targeted neuromuscular modulation reverses the asymmetry polarity (Asymmetry Index shift) and restores force vector balance, resulting in a significant Post-Activation Performance Enhancement (PAPE) in the subsequent bilateral countermovement jump. Abbreviations: CNS, Central Nervous System; RF, Rectus Femoris; EO, External Oblique; PAPE, Post-Activation Performance Enhancement. Note: This diagram represents a conceptual hypothesis based on acute sEMG findings, rather than an empirically validated multi-system mechanism.

The primary finding of this study was that introducing a minor offset load (2.5%–5% of 1RM) during the hexagonal bar deadlift (HBD) elicited a rapid, dose-dependent shift in neuromuscular drive, acutely shifting the asymmetry bias and potentiating acute power performance. Specifically, loading the non-dominant (left) side not only reversed the dominance polarity of the primary movers—shifted the Rectus Femoris Asymmetry Index from −3.85% (right-biased) to +7.41% (left-biased)—but also significantly enhanced subsequent vertical jump height (PAPE effect). Notably, individual analysis revealed a consistent directional shift across all participants, confirming that offset loading acts as a robust “stealth” stimulus capable of overriding the body’s default recruitment patterns (Figure 4). The conceptual framework illustrating how this ‘stealth’ offset loading strategy overrides the body’s default recruitment patterns to correct asymmetry and potentiate power performance is summarized in Figure 5.

### 4.1 The “hidden” asymmetry and neuromuscular redistribution

A critical observation from our baseline data was the presence of functional asymmetry even under the “Symmetric” control condition. Despite the bilateral nature of the HBD, participants displayed significantly higher activation in the dominant (right) quadriceps and erector spinae. While lacking kinetic data, this activation imbalance suggests the possibility that athletes might adopt compensatory neural strategies during bilateral closed-kinetic-chain exercises (Di Giminiani et al., 2023; Stensdotter et al., 2024; Baumgart et al., 2017).

The introduction of offset loading successfully disrupted this default pattern. Reflecting an overall increase in neural drive, the heightened demand on the weighted side forced the neuromuscular system to upregulate activation on the loaded side to maintain equilibrium. Our results align with previous findings (Saeterbakken and Fimland, 2013; Lawrence et al., 2021) in the bench press, but extend them to a multi-joint, ground-based movement. We identified a load-dependent trend (*R* ^2^ > 0.95), suggesting that coaches can “titrate” the neural drive to a specific limb with high precision using minimal load variations.

### 4.2 Contralateral core bracing: the “guy-wire” mechanism

The divergent activation pattern of the External Oblique (EO) provides mechanical insight into how the body manages offset loads. Unlike the primary movers (which activated on the loaded side), the EO exhibited a profound contralateral activation spike. For instance, loading the left side significantly increased right EO activity. This phenomenon reflects a “guy-wire” stabilization strategy, where the contralateral core musculature generates an anti-lateral flexion moment to counteract the torque created by the offset load (Kibler et al., 2012; Huxel Bliven and Anderson, 2013). This coordinated “agonist drive (ipsilateral) + core brace (contralateral)” mechanism highlights the value of offset HBD as a comprehensive training tool. It integrates limb-specific strengthening with functional core stability, a combination often lacking in traditional machine-based unilateral exercises.

### 4.3 Decoupling of effort and performance: the PAPE effect

An intriguing finding was the decoupling between subjective effort (RPE) and objective performance (CMJS). While RPE increased with offset magnitude regardless of the side, acute power performance only improved when the non-dominant (left) side was loaded. While this enhancement aligns with the timeframe of PAPE, our design does not rule out alternative explanations, such as acute alterations in motor learning, optimized intermuscular coordination, or a general psychological arousal effect induced by the novel uneven load.

We propose an “Asymmetry Modulation Hypothesis” to explain this. Loading the dominant (right) side likely exacerbated the pre-existing baseline asymmetry, potentially creating inefficient force vector transmission during the subsequent jump. Conversely, loading the non-dominant (left) side temporarily “primed” the weaker neural pathway, deliberately creating a transient overcorrection towards the non-dominant side. This acute modulation of the neural drive likely optimized the summation of forces during the bilateral CMJ, leading to the observed 8.2% increase in jump height. This suggests that the potentiation effect (PAPE) in asymmetric athletes may be maximized not by simply overloading the system, but by balancing it (Karabel and Makaracı, 2025; Xu et al., 2025).

### 4.4 Practical applications

The findings of this study offer a preliminary conceptual model for implementing offset loading in strength and conditioning. First, the “Stealth” Strategy: A mere 2.5%–5% offset is sufficient to elicit a significant neural response (>15% increase in EMG) while utilizing submaximal absolute loads. Second, Modulation of Asymmetry: For athletes with identified strength imbalances, offset HBD serves as a potent corrective exercise. The linear AI shift indicates that practitioners can progressively adjust the offset magnitude to match the athlete’s specific degree of asymmetry. Finally, Potentiation Complexes: Loading the non-dominant side prior to explosive movements may serve as an effective localized warm-up or contrast set to enhance bilateral power output.

### 4.5 Limitations

Several limitations should be noted. First, the sample consisted of young, resistance-trained males with right-side dominance; results may differ in left-dominant individuals or female athletes. Second, this was an acute study; longitudinal research is needed to determine if these acute neural adaptations translate to chronic strength gains or persistent asymmetry modulation. Third, asymmetry was quantified solely *via* sEMG. The lack of dynamic kinetic data (e.g., dual force plates) limits our ability to confirm how these altered activation patterns translate to actual ground reaction forces. Fourth, normalizing EMG to %MVIC presents inherent limitations, particularly for postural and stabilizing muscles like the External Oblique and Erector Spinae. The multi-planar stabilizing functions of these muscles during a dynamic deadlift are not fully captured by conventional isometric tests; thus, the reported activation levels should be cautiously interpreted as indicators of task-specific involvement rather than absolute increases in neural drive. Fifth, the absence of kinematic data represents a key limitation that directly constrains the interpretation of the proposed postural and stabilization mechanisms, and future research should investigate kinematic changes to ensure that offset loading does not compromise lifting technique or safety. Finally, although a force platform was used, CMJ height was calculated *via* flight time rather than the more robust impulse-momentum method. This may limit the sensitivity of our jump assessment in capturing minute kinetic changes related to PAPE.

## 5 Conclusion

In conclusion, the introduction of minor offset loads (2.5%–5% of 1RM) during the hexagonal bar deadlift serves as a potent and precise “stealth” stimulus that effectively overrides the body’s habitual recruitment patterns. We identified that even under symmetric loading conditions, athletes exhibit a functional bias toward their dominant limb. Offset loading applied to the non-dominant side successfully acutely shifts this activation bias in a load-dependent manner (*R* ^2^ > 0.95), achieving a consistent individual modulation across our cohort.

Crucially, this study highlights a functional decoupling between subjective effort and objective performance. While offset loading increases perceived exertion, targeting the non-dominant limb is associated with a significant enhancement of post-activation vertical jump height. This supports our “Asymmetry Modulation Hypothesis,” suggesting that optimal power expression in asymmetric athletes is achieved not merely by increasing total load, but by optimizing the balance of force transmission.

From a practical standpoint, strength and conditioning coaches and rehabilitation specialists can utilize this preliminary conceptual model to help modulate neuromuscular drive to a specific limb while utilizing submaximal absolute loads and avoiding complex unilateral setups. We recommend incorporating offset HBD as a strategic tool in both corrective phases and pre-competition potentiation protocols, specifically targeting the non-dominant side to maximize bilateral performance efficiency.

## Data Availability

The raw data supporting the conclusions of this article will be made available by the authors, without undue reservation.
